# A metric learning method for estimating myelin content based on T2-weighted MRI from a de- and re-myelination model of multiple sclerosis

**DOI:** 10.1371/journal.pone.0249460

**Published:** 2021-04-05

**Authors:** Glen Pridham, Shahnewaz Hossain, Khalil S. Rawji, Yunyan Zhang

**Affiliations:** 1 Department of Clinical Neurosciences, University of Calgary, Calgary, Alberta, Canada; 2 Hotchkiss Brain Institute, University of Calgary, Calgary, Alberta, Canada; 3 Department of Medical Sciences, University of Calgary, Calgary, Alberta, Canada; 4 Department of Radiology, University of Calgary, Calgary, Alberta, Canada; Instituto Cajal-CSIC, SPAIN

## Abstract

Myelin plays a critical role in the pathogenesis of neurological disorders but is difficult to characterize in vivo using standard analysis methods. Our goal was to develop a novel analytical framework for estimating myelin content using T2-weighted magnetic resonance imaging (MRI) based on a de- and re-myelination model of multiple sclerosis. We examined 18 mice with lysolecithin induced demyelination and spontaneous remyelination in the ventral white matter of thoracic spinal cord. Cohorts of 6 mice underwent 9.4T MRI at days 7 (peak demyelination), 14 (ongoing recovery), and 28 (near complete recovery), as well as histological analysis of myelin and the associated cellularity at corresponding timepoints. Our MRI framework took an unsupervised learning approach, including tissue segmentation using a Gaussian Markov random field (GMRF), and myelin and cellularity feature estimation based on the Mahalanobis distance. For comparison, we also investigated 2 regression-based supervised learning approaches, one using our GMRF results, and another using a freely available generalized additive model (GAM). Results showed that GMRF segmentation was 73.2% accurate, and our unsupervised learning method achieved a correlation coefficient of 0.67 (top quartile: 0.78) with histological myelin, similar to 0.70 (top quartile: 0.78) obtained using supervised analyses. Further, the area under the receiver operator characteristic curve of our unsupervised myelin feature (0.883, 95% CI: 0.874–0.891) was significantly better than any of the supervised models in detecting white matter myelin as compared to histology. Collectively, metric learning using standard MRI may prove to be a new alternative method for estimating myelin content, which ultimately can improve our disease monitoring ability in a clinical setting.

## 1 Introduction

Myelin plays an important role in maintaining neurological functions. It can enhance signal conduction speed directly by 20–100 times, and provides critical neuroglial support for the underlying axons [1]. The integrity of myelin is implicated in normal development, ageing, and numerous pathological conditions of the nervous system such as multiple sclerosis (MS) [2]. Thus the availability of a non-invasive method to reliably characterize myelin content is critical.

There have been significant effort in deriving myelin assessment methods previously using magnetic resonance imaging (MRI) techniques. Example measures include: myelin water fraction [3, 4], quantitative T1 or T2 [3–5], radial diffusivity [4, 6], and magnetization transfer [3–5, 7]. An alternative approach is assessing the distribution pattern (texture) of MRI pixels. Image texture refers to the unique inter-pixel relationships associated with a specific tissue. In MRI, such relationships highlight the intrinsic biophysical property of the underlying tissue as reflected by its signal intensity and relaxation [8]. Therefore, macroscopic analysis of MRI texture provides an integral estimation of the biochemical properties of tissue microstructure as done in histology. Combining with pertinent characterization techniques, texture analysis may enable robust evaluation of myelin content using myelin-sensitive MRI. Indeed, texture analysis of T2-weighted MRI has shown considerable potential to detect changes in myelin integrity using various methods: first and second order statistics [9], directional statistics [10, 11], or spectral analysis [12], although their sensitivity and specificity require further verification.

Analysis of MRI texture also includes methods that combine parameter estimation with tissue classification, such as Markov random fields (MRFs) [13, 14]. In a MRF, image texture is modelled as a local interaction between neighbouring pixels. The outcome of a MRF classification model can directly serve as input for subsequent regression, or for latent variable analysis based on Mahalanobis distances [15], if the MRF is Gaussian (GMRF). Mahalanobis distance is a measure of the distance between a point and a distribution, characterized by the number of standard deviations that a point is away from the mean of a distribution. It takes account the covariance between variables in multi-dimensional measures and is scale invariant. Co-registering the Mahalanobis metric space to a physically meaningful metric space, such as histological myelin density, may allow direct, unsupervised prediction of tissue microstructural properties. Notably, advances in machine learning technologies over the past 20 years [16] have made supervised training of statistical models highly attractive, particularly given the availability of robust non-linear image co-registration algorithms [17, 18]. Supervised learning is also typically easier to conduct and may perform better than unsupervised learning [19], but the ground truth is not always available [20].

In this study, we proposed an unsupervised learning approach for predicting histological myelin, and its counter-staining tissue, cellularity, based on T2-weighted MRI acquired from a mouse model of de- and re-myelination of MS [21, 22]. Feature estimation used the Mahalanobis distance, following GMRF segmentation. Subsequently, we compared the derived MRI features to histology, and to results from two supervised regression models trained using histological myelin and cellularity standards; one model based on our GMRF segmentation results. To ensure accuracy, we also compared segmentation between our GMRF and an established MRF [14].

## 2 Materials and methods

This study was approved by the Health Sciences Animal Care Committee at the University of Calgary (ID: AC13-0246). During animal model creation, imaging, and tissue sampling, anesthetic agents including ketamine and xylazine were used to ensure optimal comfort of the animals and quality of the data.

### 2.1 Animal model

The source data used in this experiment have been previously reported in another study [12]. In brief, we examined 18 female C57BL/6 mice aged 8–10 weeks (Charles River, Quebec, Canada). Demyelinated lesions were induced by an expert (KSR) to the ventral white matter of thoracic spinal cord (T3/4) using a chemical toxin, lysolecithin. This model featured peak demyelination at 7 days post lysolecithin injection, active remyelination at 14 days, and nearly complete recovery by 28 days, as established in various studies [3, 21, 22].

### 2.2 MR imaging

Cohorts of 6 mice underwent MRI at each time-point (7, 14 and 28 days), totaling 18 animals. Image acquisition used a 35-mm-diameter volume coil at our 9.4T animal scanner (Bruker Biospin, Germany), following head and limb fixation with dedicated tapes and intraperitoneal injection of anesthesia (100 mg/kg ketamine/xylazine) immediately before MRI to keep the animals still. Throughout the experiment, the animals were under respiratory gating, and temperature and heartrate monitoring. The MRI protocol included a RARE T2 sequence with the following parameters: TR/TE = 2500/15 ms, echo train length = 8, slice thickness = 0.75 mm for 7 slices, field of view = 1.5x1.5 cm^2^, matrix = 256x256, and pixel size = 0.06x0.06 mm. As lesion induction was stereotactically controlled at a single injection site in each animal, we focused only on the MRI slice at the centre showing the largest lesion area.

### 2.3 Histology procedure

Post imaging, the animals were sacrificed immediately through transcardial perfusion with 4% paraformaldehyde. The thoracic spinal cords were then sampled and prepared for histology. Specifically, 20 μm-thick transverse slices were cut using a cryostat centered at the lesion, totaling 10–80 sections per animal to ensure complete coverage of the thickness of an individual MRI slice at the location (750 μm-thickness). The sections were stained with eriochrome cyanine for myelin, and neutral red counter stain for nuclei/cellularity [23], and digitalized using a confocal microscope (Olympus BX51, Japan). The section with the largest lesion area in each animal was used for MRI correspondence. Overall, 4 of the 18 animals were excluded from statistical analysis: 3 were sliced longitudinally (one per time-point), and one (1) did not show a MRI-matching lesion (mouse 4).

### 2.4 Image pre-processing for MRI and histology

Image processing was primarily performed using R, version 3.4.3 [24], via the EBImage package [25]. For histological images, we first manually removed background artifacts and loose tissue strands using Adobe Photoshop CC (2017.1.0). Then, we separated the myelin and cell nuclei staining as blue and red channels using R, and performed image normalization in 2 steps: pixel-wise, which divided each pixel value in a channel by the total pixel intensity across colors, and channel-wise, to the range [0, 1]. Standardization of MRI included masking of the spinal cord using ImageJ 1.5 [26] and intensity normalization to [0, 1].

**Fig 1:**
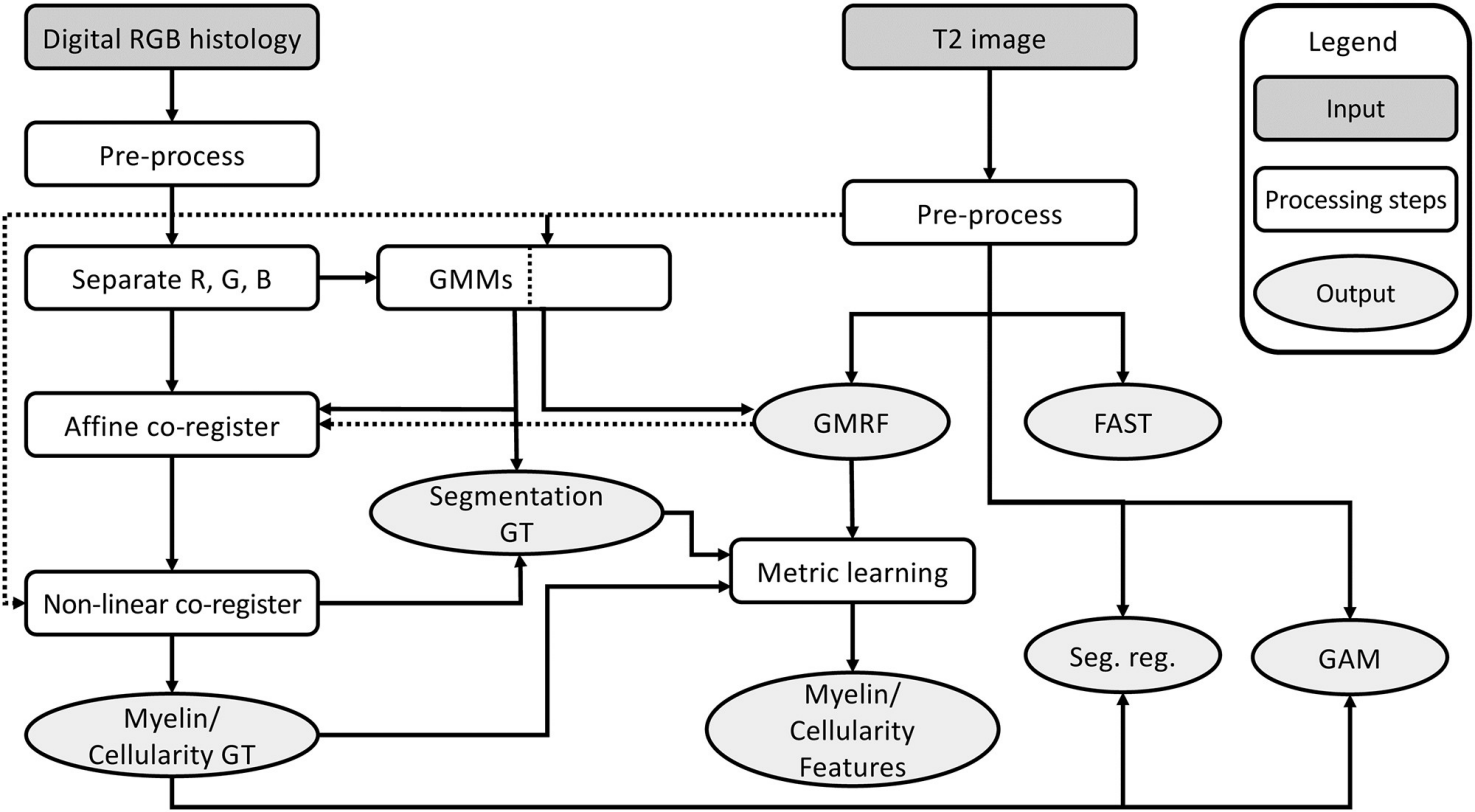
Image processing and analysis pipeline. Shown are the main steps involved in this study for image preparation, MRI and histology alignment, and myelin and cellularity estimation using MRI. Note: RGB: red, green, blue; Myelin/cellularity GT: myelin and cellularity value ground truth; GMM: Gaussian mixture model; Segmentation GT: segmentation ground truth of histology; GMRF: Gaussian Markov random field; Seg. reg: segmentation regression; GAM: generalized additive model.

The next step was image co-registration. This included primarily affine transformations [18] to align the longitudinal images in both MRI and histology, and to align histology and MRI cross-sectionally per subject. For the latter, we first embedded individual histological images in a white background such that each had a 4096x4096 dimension, and then down sampled them to 64x64 to match the scale of MRI using the Gaussian pyramid approach [27]. Subsequently, we segmented the MRI and histology images and affine co-registered the segmentation results from histology to MRI [18]. To further improve their alignment, we conducted a second, non-linear co-registration [17]. This co-registration included a pre-processing step that regressed MRI signal intensity against affine co-registered histology density using a random forest approach [28]. The purpose of the random forest was to linearize the relationship between MRI and histology to enhance the quality of co-registration (Fig 1).

### 2.5 Texture analysis

**Fig 2:**
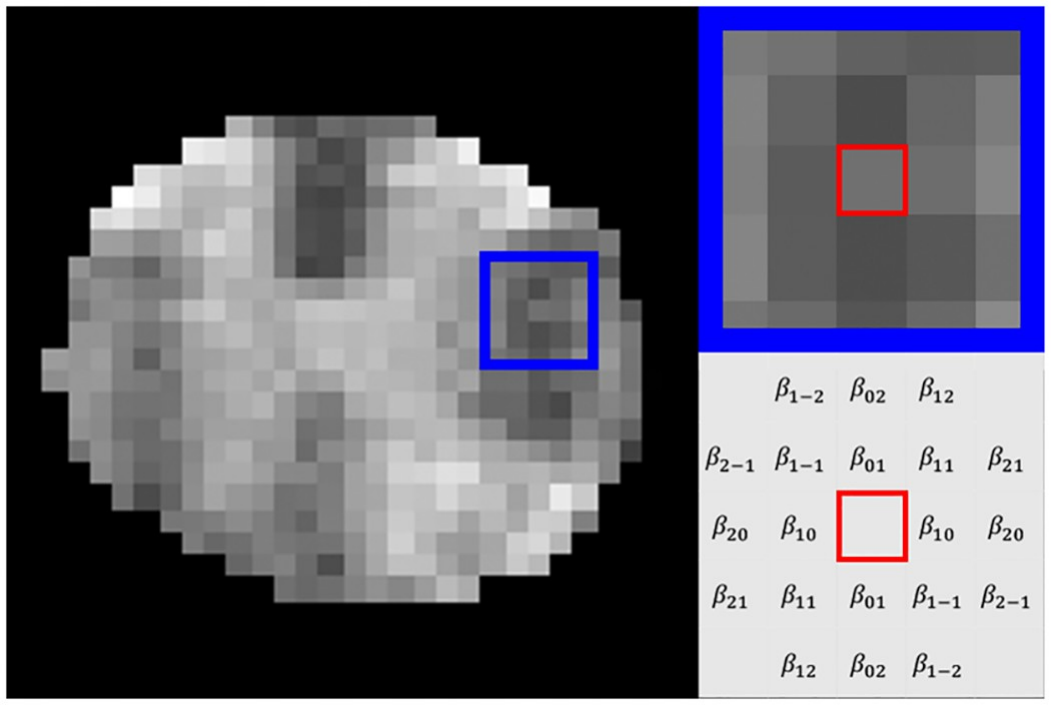
Markov random field model. Shown are an example T2-weighted MR image from a mouse spinal cord (left), a focal highlight in the left lateral white matter sized 5x5 pixels (blue square, left and top right), and the Markov model parameters for an arbitrarily selected pixel within the blue (red square, bottom right). The pixel was modelled as a function of its 20 nearest neighbours, with outcomes scaled by the 10 unique, symmetric parameters.

For both supervised and unsupervised approaches, we incorporated local textural information, derived from the pairwise relationships between an individual pixel and its neighbours, namely, Markov texture. Our neighbourhood structure, N, was symmetric about the central pixel and was sorted by radial distance then by angle with respect to the x-axis (Fig 2). In this study, we chose 20 symmetric neighbours, which gave rise to 10 unique parameters. This choice had shown to be highly effective previously in discriminating natural textures [29].

### 2.6 Unsupervised segmentation

The segmentation process seeks to convert a set of random variables, $\left\{{\overset{⃑}{x}}_{i}\right\}$, such as pixel intensity values in an image, into a hidden label membership field *Z*_*il*_, defined by:

$$Z_{il}=\{\begin{array}{ll} 1 & \text{if}\;x_{i}\in \;\text{label}\;l\;\\ 0 & \text{otherwise} \end{array}$$ (1)

Based on histopathology, we *a priori* identified 4 major segmentation groups: background (BG), white matter (WM), grey matter (GM), and cell body (CB). In particular, we combined the substantia gelatinosa (SG) component with induced lesions to form the CB group, given their similarity in staining properties. For MRI, we included a 5^th^ label, partial volume (PV), to account for the potential partial volume effect between WM and GM [30]. In this study, we used a Gaussian Mixture Model (GMM) to segment histology images, and our customized GMRF to segment MRI, with the latter further compared with a different MRF-based open-source algorithm (FAST, Oxford, UK) as described below (Section 2.6.2).

#### 2.6.1 Segmentation with GMMs

GMMs segment data into sub-populations with Gaussian statistics. We used GMMs for two purposes. First, segment the color histology images into 4 tissue types (BG, WM, GM, and CB), to generate the ground truth for validating MRI segmentations; calculate tissue means; and perform receiver operator characteristic (ROC) curve analysis. Second, initialize the GMRF segmentation by preliminary segmenting the MRI into 5 labels using T2 signal intensity alone. All GMM estimations were performed using the Mclust algorithm with default prior, which automatically selected the best model using the Bayesian information criterion [31].

#### 2.6.2 Segmentation with an open-source MRF

The FAST algorithm implemented in the open-source FSL library is a common method for tissue segmentation in neuroimaging [14], which uses a MRF akin to our GMRF model. For comparison, we used the R implementation [32] of FAST. Using the best recommended parameter values, the FAST segmented MR images into 3 options: BG/CB/GM/PV/WM, BG/CB/GM/WM, and BG/GM/WM. Based on our preliminary results, the FAST segmentation was not as accurate as our GMRF, so metric learning analysis used the latter only; our final results also confirmed the lack of competency of FAST in the current study (Table 1).

**Table 1:** Segmentation confusion matrices (N = 6307).

		GMRF			FAST		
		CB	GM	WM	CB	GM	WM
**Histology**	**CB**	11‰	25‰	25‰	31‰	15‰	15‰
	**GM**	50‰	270‰	129‰	236‰	157‰	56‰
	**WM**	5‰	34‰	451‰	27‰	126‰	338‰

‰: per mille.

### 2.7 Unsupervised segmentation and metric learning with a GMRF

#### 2.7.1 GMRF theory

Given a vector of image intensities: $\overset{⃑}{x}$, and a possibly non-constant mean, $\overset{⃑}{\mu }$, any GMRF can be written as:

$$p(\vec{x})={\left|\frac{1}{2\pi }Q\right|}^{\frac{1}{2}}\text{exp}(-\frac{1}{2}{\left(\vec{x}-\vec{\mu }\right)}^{T}Q(\vec{x}-\vec{\mu }))$$ (2)

with inverse covariance *Q* [33]. Note that *Q*_*ij*_ = 0 if *x*_*i*_ and *x*_*j*_ are not neighbours and are therefore conditionally independent [33]. We hypothesized that tissues were classifiable based on their differences in $\overset{⃑}{\mu }$ and *Q*. This also implied mathematically that these variables are hidden-label-dependent. We used the following parameterization:

$$\mu_{i}=\sum_{l=1}^{N_{l}}Z_{il}{\overset{´}{\mu }}_{l}$$

$${\overset{´}{\mu }}_{l}=\frac{1}{\sum_{i=1}^{\left|\vec{x}\right|}Z_{il}}\sum_{i=1}^{\left|\vec{x}\right|}Z_{il}x_{i}$$

$$Q_{\left(i,j)(i^{\prime },j^{\prime }\right)}\equiv 0\;\text{if}\;x_{\left(i,j\right)}\;\text{or}\;x_{\left(i^{\prime },j^{\prime }\right)}\in \text{background}\;(\text{exclusive})$$

$$Q_{\left(i,j)(i^{\prime },j^{\prime }\right)}=\left\{ \begin{array}{c} -\frac{1}{2}\sum_{l=1}^{N_{l}}\frac{Z_{(i,j)l}\beta_{l,i-i^{\prime }j-j^{\prime }}+Z_{(i^{\prime },j^{\prime })l}\beta_{l,i^{\prime }-i\;j^{\prime }-j}}{\beta_{l,00}}\\ 0 \end{array}\begin{array}{l} ,\;\text{for}\;\text{neighbours}\\ ,\;\text{otherwise} \end{array}\right.$$

$$Q_{\left(i,j)(i,j\right)}=\overset{N_{l}}{\underset{l=1}{\sum }}\frac{Z_{(i,j)l}}{\beta_{l,00}}$$ (3)

where (*i*, *j*) denotes image indices before vectorization.

#### 2.7.2 Hidden label field estimation/GMRF segmentation

Parameter estimation with the GMRF included 2 steps: 1) estimating the hidden (tissue) label field (GMRF segmentation), and then 2) estimating the parameters for each associated tissue label. These steps iterated until convergence. The latter was done using least-squares as loss functions [29], with the background label fixed for optimal segmentation at: *μ* = 0, *β*_00_ = 10^−30^, and *β*_*ij*_ = 0.

We used results from the GMMs to initialize our GMRF segmentation for T2-weighted MRI as described above. Iterative refinement of the label field took a classical approach known as Gibbs sampling achieved pixel-wise, using the simulated annealing technique [34, 35]. Specifically, using an arithmetic coding structure [36], we iteratively updated the label field of all non-neighbouring pixels according to the proposal distribution (S1 File):

$$p{\left(Z_{il}=1|Z_{-il},\vec{x},Q,\vec{\mu }\right)}^{t^{-1}(n)}\approx \frac{1}{\Pi }p{\left(x_{i}|N_{i},Q,\vec{\mu },Z_{il}=1,{\text{Z}}_{-\text{il}}\right)}^{\;t^{-1}(n)}p{\left(Z_{il}=1\right)}^{t^{-1}(n)}$$ (4)

where {−*i*} is the set of pixels excluding the *i*th one. This distribution allowed to calculate the probability of a pixel belonging to any associated label and then randomly sample a label for the pixel based on its probability. According to Eq (3), the first term on the right-hand-side of Eq (4) is:

$$p(x_{i}|N_{i},Q,\vec{\mu },Z_{il}=1,{\text{Z}}_{-\text{il}})=\sqrt{\frac{1}{2\pi \beta_{l,00}}}\text{exp}(-\frac{1}{2\beta_{l,00}}{\left(x_{i}-\mu_{i}-J_{i}(Q)\beta_{l,00}\right)}^{2})$$ (5)

where *J*_*i*_ ≡ −∑_*B* = {−*i*}_(*Q*_*iB*_(*x*_*B*_ − *μ*_*B*_)). To validate the fitting results of our GMRF model, we also performed simulation via Gibbs sampling (S2 File) using Eq (5).

Next, the probability of each label pixel was set *a priori* to:

$$p(Z_{il}=1)=\gamma_{l}$$ (6)

where *$\overset{⃑}{\gamma }={\left[0.010,0.045,0.030,0.061,0.854\right]}^{T}={\left[CB,GM,PV,WM,BG\right]}^{T}$*. These values were estimated from the median of the day 28 MRI scans that were essentially lesion free. Fixing $\overset{⃑}{\gamma }$ allowed our algorithm to have *a priori* knowledge, which discouraged biologically unlikely outcomes to have high likelihood values (e.g CB group).

Finally, the temperature at the *n*th iteration, t(n), was set using the heuristic schedule, which had the ability to converge to a physically realistic and stable solution [13, 35]:

$$t(n)=\{\begin{array}{c} \frac{{\text{log}}_{\text{e}}(4)}{{\text{log}}_{\text{e}}(3+n)}\\ 0 \end{array}\;\begin{array}{l} \text{for}\;n\leq 253\\ \;253<n<259 \end{array}$$ (7)

where the number of iterations was determined by the condition that the final temperature be $t_{f}=0.25$. When the temperature was 0 we picked the maximum of Eq (7), making it equivalent to iterated conditional modes [34], where the most likely label was picked at each iteration. MR images were initially segmented into 5 labels. When comparing to a segmentation with fewer labels (e.g. histology), we removed obsoleted labels (e.g. PV) and re-normalized Eq (4).

#### 2.7.3 Myelin and cellularity feature estimation

Our metric learning process enabled feature estimation. It involved constructing a metric space for MRI based on the output of GMRF segmentation, and then co-registering the MRI metric space to the corresponding histological metric space. The co-registration used tissue means as landmarks, which were estimated using our down-sampled GMM segmentation of histology (literature standards could be used here instead). For the MRI metric, we computed the Mahalanobis distance (*d*) between each pixel and the mean of each label as: *d*_*WM*_, *d*_*GM*_, and *d*_*CB*_. Each of these Mahalanobis distances was scaled by a constant optimized by Nelder-Mead, which gave the minimal root-mean-squared distance between tissue means of the MRI and histology metric spaces. The scaled distances were then used for numerical (Nelder-Mead) multilateration, which enabled co-registering the MRI-space position (WM, GM, CB) to the histology-space position (myelin, cellularity), and the histology-aligned MRI space positions for myelin and cellularity became our correspondent MRI features, respectively. As this process required at least three reference points or tissue groups, all of our segmentations contained three or more labels. The Mahalanobis distance of the *i*th pixel from the *l*th tissue mean was calculated using the mean, *μ*_*l*_, variance, *β*_*l*,00_, neighbourhood parameters, *β*_*l*,*j*_ and neighbour means *μ*_*j*_ as:

$$d_{il}=\sqrt{\frac{1}{\beta_{l,00}}{\left(\left(x_{i}-\mu_{l})-\underset{j\in \{N_{i}\}}{\sum }\beta_{l,j}(x_{j}-\mu_{j}\right)\right)}^{2}}$$ (8)

### 2.8 Supervised learning

Using histology results as the ground truth, we considered two non-linear regression models: segmentation regression, based on outputs from our GMRF segmentation, and a black-box generalized additive model (GAM). Regarding the latter, we also included a linear regression as the simplest type of GAM (linear GAM), for quantitative comparison. Furthermore, we considered two loss functions for each model: root-mean-squared error (RMSE) and mean-absolute-error (MAE). Where not explicitly stated, we refer to MAE. Finally, we explored the impact of focal lesions by explicitly including a manual lesion mask, where for optimal reliability, we focused only on the four day 7 mice because they had the largest lesions.

#### 2.8.1 Segmentation regression

We extended our GMRF segmentation algorithm to include a response variable, the staining density of myelin/cellularity, and then used the GMRF outcomes and MRI signal intensity as predictors of the response. For the RMSE loss, we used a linear random effects model [37], while for the MAE, we performed median regression independently for each segmented label [38]. Our final model included the manual lesion masks as a new label using the modified GMRF segmentation achieved by overriding existing labels.

#### 2.8.2 Generalized Additive Models (GAMs)

GAMs can capture non-linearities by allowing arbitrary relationships between predictor variables. Each observation *y*_*i*_ takes the form [16]:

$$y_{i}=\beta_{0}+\beta_{1}f({\vec{x}}_{i})+\epsilon_{i}$$ (9)

where the unknown function, *f*, must be expanded along a user-specified basis. The linear model (linear GAM) represents the identity case *f*(*x*) = *x*. Our other GAM models used a thin-plate spline with the default number of knots [39, 40]. To understand the impact of predictors on our blue and red histology standards, we performed step-wise regression using: 1) T2 signal intensity alone (Markov- GAM), 2) T2 signal intensity plus texture (Markov GAM), and then 3) adding the manually identified lesion masks as a binary dummy variable, *l*, to #2 (Markov+ GAM). The Markov texture here also used a 20-neighbourhood setting per pixel to ensure comparability with our GMRF-based approach. The full GAM model was:

$$y_{i}=\beta_{0}+\beta_{1}f_{1}(x_{i}(1-l_{i}))+\beta_{2}f_{2}(x_{i}l_{i})+\underset{j\in \{N_{i}\}}{\sum }\beta_{j}f_{j}(x_{i}(1-l_{i}),x_{j}(1-l_{i}))+\epsilon_{i}$$ (10)

### 2.9 Time cohort analysis

To understand how MRI estimates performed over time, we conducted a group-level analysis at each time-point for all three learning approaches and compared that with the corresponding histology. This was done without inclusion of lesion masks. The estimated outcomes for each learning method were combined per time-point after initial longitudinal co-registration of the source images. For both MRI and histology, the median maps per time-point were calculated.

### 2.10 Model validation and statistical analysis

All models were validated using the 14 mice with corresponding histology. Unsupervised approaches were assessed directly using Pearson correlation. Supervised approaches were leave-one-out cross-validated, with error estimated by the standard deviation. In addition, pairwise comparisons used the Mann-Whitney test, and segmentation accuracies used the binomial test. To further test the ability of our unsupervised MRI myelin feature versus regression, ROC curve analysis was performed [41], using the co-registered histology myelin segmented in the WM as ground truth. Comparison of the area under the ROC (AUC) curves used the Delong test. All statistics were computed using R [24]. A p < 0.05 was considered significant. All reported confidence intervals were 95%. Members in the background label in either histology or MRI were excluded in calculating summary statistics.

## 3 Results

### 3.1 Segmentation outcomes

#### 3.1.1 Qualitative results

**Fig 3:**
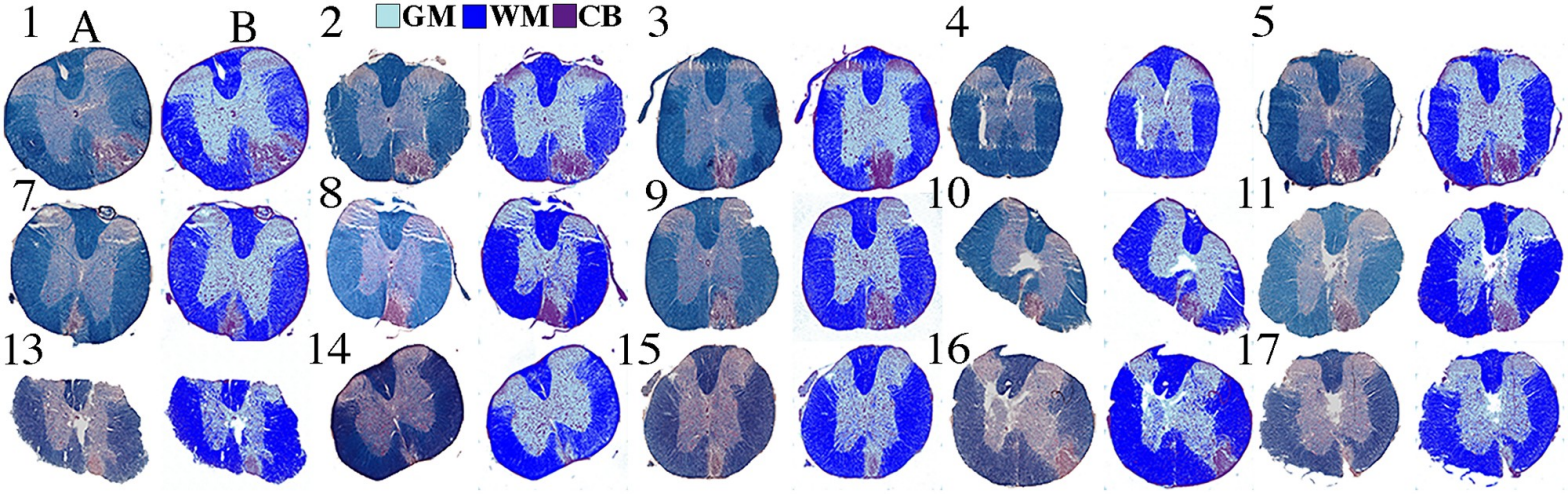
Histology segmentation. Shown are the original histological images (A) obtained from each animal (numbers), and the corresponding segmented images (B) using the red-green-blue intensity. Row indicates time cohort: day 7 (row 1), day 14 (row 2) or day 28 (row 3). Note: mouse 4 did not show a convincing lesion in histology to match MRI so was excluded from correlative analyses.

**Fig 4:**
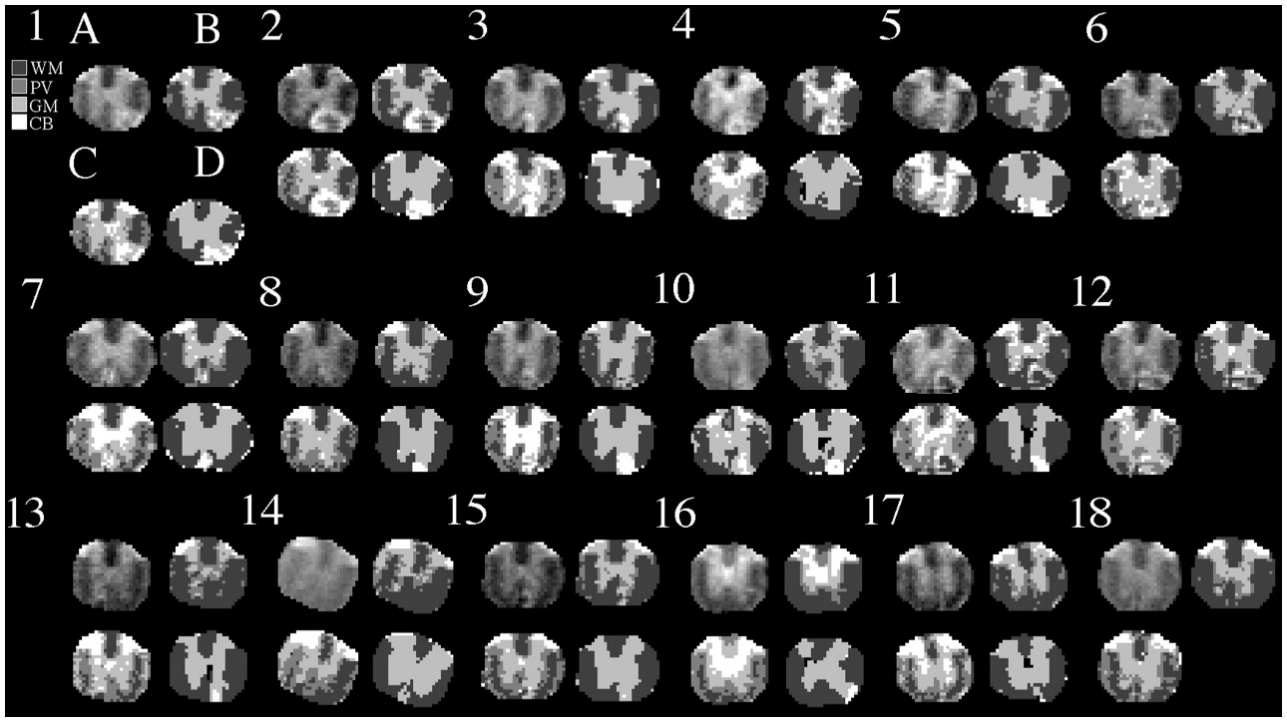
MRI segmentation. Shown are the original T2 MRI images (A) from each animal (numbers), the corresponding segmentation results of MRI using our GMRF (B) and the open-source software FSL FAST (C), and the associated histology segmentation using our GMM method for comparison (D). Row indicates time cohort: day 7 (row 1), day 14 (row 2) or day 28 (row 3).

Overall, we analyzed the MRI of all 18 animals, and MRI versus histology of 14 animals. Visually the GMM segmentation for histology was consistent with the expected anatomy (Fig 3). In MRI, our GMRF segmentation appeared better than the FAST segmentation in all animals. The GMRF correctly identified more tissue, and had smoother and more continuous labels for the main tissues, such as WM and GM, than FAST (Fig 4). The segmentation performance was the best for MR images that had relatively good visual contrast.

#### 3.1.2 Quantitative results

Based on the GMM segmentation of histology, the estimated tissue mean for myelin (blue) across all animals was (mean ± sd): 0.735 ± 0.119 (WM), 0.333 ± 0.117 (GM), and 0.341 ± 0.139 (CB). For cellularity (red), they were: 0.230 ± 0.113 (WM), 0.666 ± 0.120 (GM), and 0.785 ± 0.175 (CB) respectively.

Excluding background pixels, the GMRF segmentation was 73.2% (CI: 72.1%-74.3%) accurate for MRI, significantly greater than both FAST [52.6% (CI: 51.3–53.8%)] and the initial GMM [58.6% (CI: 57.4–59.8%)]. Tissue-wise, the FAST appeared to be more specific to CB than the GMRF, showing 2.8 times as many true positive CB labels. However, the FAST also had 4.7 times as many false positive CB due to mislabeling of GM (Table 1). Overall, the total number of CB pixels was small (382/6307 = 6.1%) as compared to the WM (3092/6307 = 49.0) and GM (2833/6307 = 44.9%).

**Fig 5:**
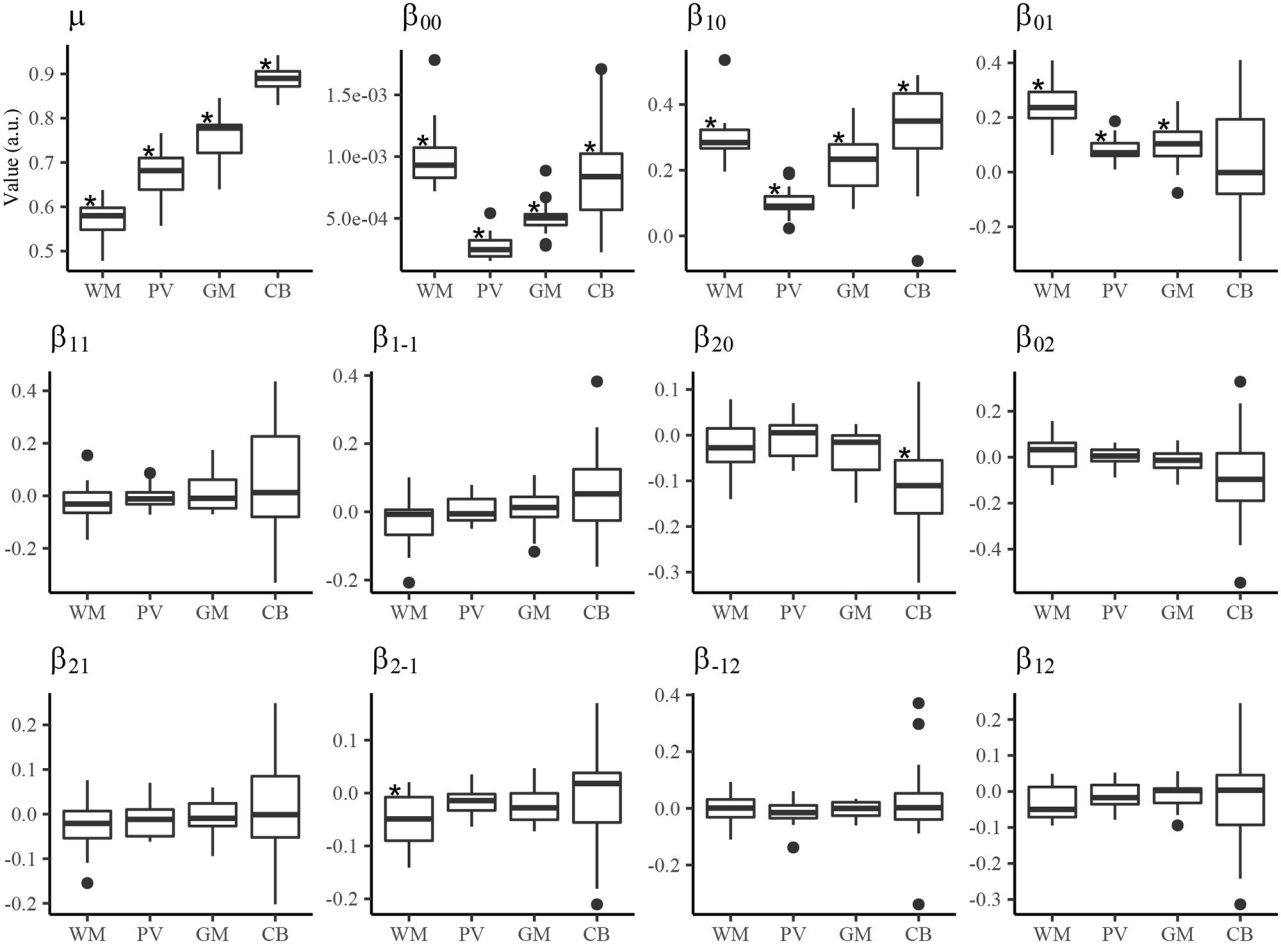
GMRF MRI segmentation parameters. Shown are boxplots of the GMRF segmentation parameters across all subjects. Parameters showing no overlap of the inter-quartile ranges between tissues (WM, PV, GM, CB) suggest tissue-dependent and approximately independent of the test subject. The symbol, ⋆, indicates parameters significantly different from zero based on Bonferroni-corrected Wilcox test. Note: WM: white matter; PV: partial volume; GM: gray matter; CB: cell body.

Out of the 12 texture parameters in the GMRF model, 4 were significantly different from zero (Fig 5): the mean (*μ*), conditional variance (*β*_00_) and the first 2 of the 10 neighbouring parameters (*β*_01_ and *β*_01_). Moreover, using the parameters generated by the GMRF segmentation (S1 Table), our simulated images showed a high similarity to the initial MRI in all but one animal (mouse 14), although the simulation showed a notably pixelated appearance (S1 Fig).

### 3.2 Estimation outcomes for the myelin feature

#### 3.2.1 Visual inspection

**Fig 6:**
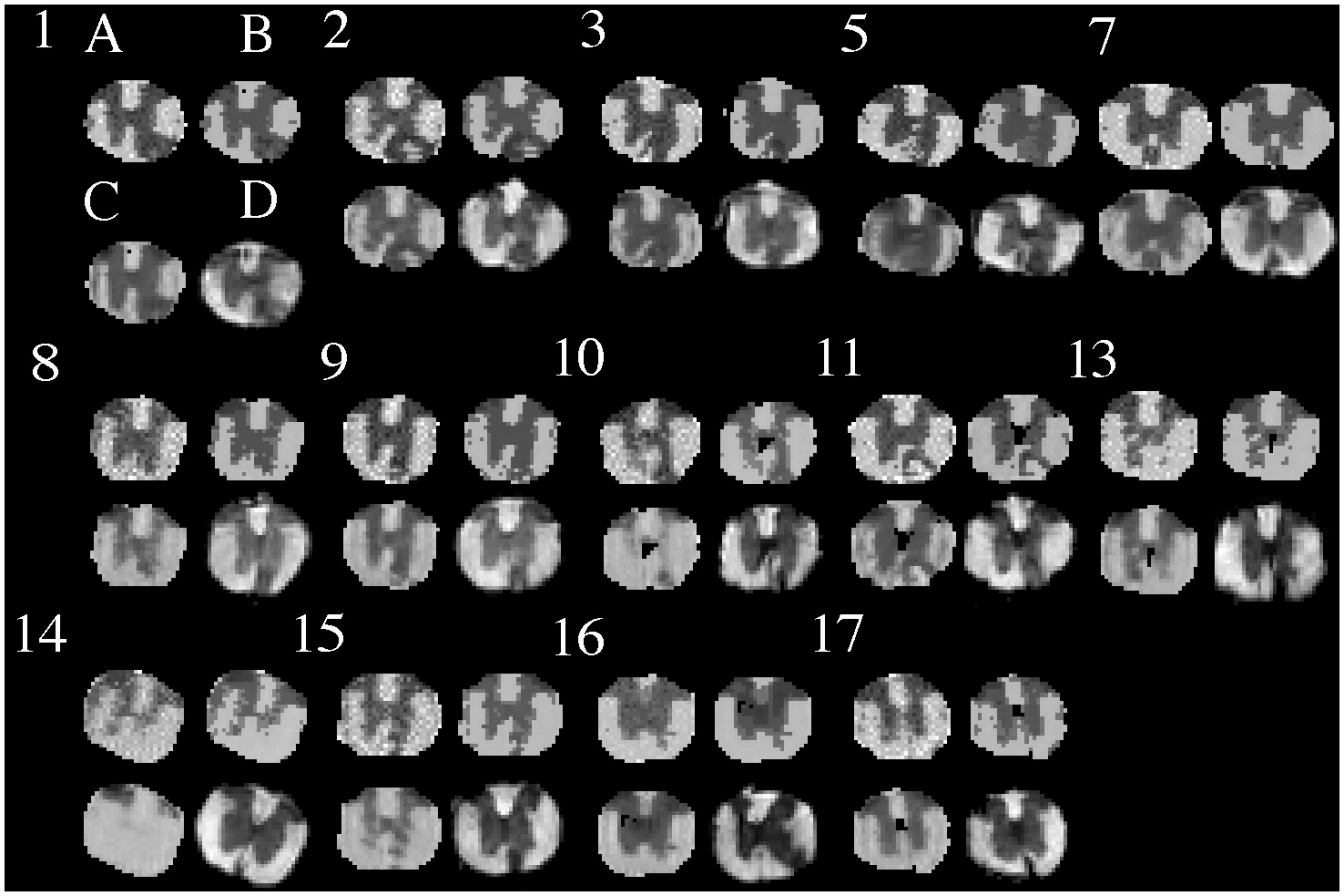
Myelin predictions. Shown are the myelin feature images learned from the MRI of the 14 mice that have histological correspondence using: our GMRF (A), segmentation regression associated with our GMRF (B), Markov GAM using a freely available software (C), and the histological standard (D). Note: there is no clear myelin/WM structure detected using Markov GAM in mice 10 and 14, and the lesion cores appear to be overestimated in mice 2 and 10 in the MRI methods.

The estimated myelin content of GM and WM was consistent with the histological findings from all methods, except for mice with poor MRI contrast: 13 and 14, or with large lesion cores: 2, 11 and 16 (Fig 6). Compared to histology segmentation, there appeared to be a systematic under-fit in MRI models, especially using RMSE, as indicated by the residual correlations (Table 2), and residual images (S2 Fig). In addition, the segmentation regression and the Markov GAM outcomes appeared smoother and with smaller lesion core discrepancies than the unsupervised GMRF features, with the segmentation regression predictions having less intra-mouse variability than the GAMs (e.g. mouse 14). Between loss functions, all MAE regressions showed better contrast than RMSE regressions in supervised learning (S3 and S4 Figs).

**Table 2:** Mean ± standard deviation (min to max) cross-validated error and 95% confidence interval (min to max, last row) of myelin model accuracies.

Model	Loss	R2	Pearson Correlation	Residual Correlation
Linear GAM	RMSE	0.36 ± 0.17	0.68 ± 0.11	0.83 ± 0.05
		(−0.05 to 0.60)	(0.47 to 0.85)	
	MAE	0.36 ± 0.21	0.68 ± 0.11	0.76 ± 0.07
		(−0.17 to 0.64)	(0.47 to 0.85)	
Segmentation Regression	RMSE	0.46 ± 0.16	0.70 ± 0.12	0.71 ± 0.10
		(0.17 to 0.70)	(0.43 to 0.84)	
	MAE	0.43 ± 0.18	0.70 ± 0.11	0.55 ± 0.15
		(0.06 to 0.72)	(0.43 to 0.85)	
Segmentation Regression+	RMSE	**0.59 ± 0.05**	0.78 ± 0.04	0.60 ± 0.09
		(0.51 to 0.64)	(0.73 to 0.81)	
	MAE	0.57 ± 0.05	0.78 ± 0.04	**0.41 ± 0.13**
		(0.50 to 0.61)	(0.73 to 0.80)	
Markov- GAM	RMSE	0.39 ± 0.18	0.69 ± 0.11	0.81 ± 0.07
		(0.00 to 0.64)	(0.45 to 0.86)	
	MAE	0.37 ± 0.22	0.69 ± 0.11	0.70 ± 0.11
		(−0.11 to 0.66)	(0.44 to 0.85)	
Markov GAM	RMSE	0.41 ± 0.20	0.69 ± 0.12	0.75 ± 0.10
		(−0.09 to 0.63)	(0.37 to 0.84)	
	MAE	0.40 ± 0.22	0.70 ± 0.12	0.70 ± 0.11
		(−0.13 to 0.67)	(0.39 to 0.85)	
Markov GAM+	RMSE	0.58 ± 0.11	0.78 ± 0.08	0.59 ± 0.14
		(0.43 to 0.67)	(0.67 to 0.84)	
	MAE	0.58 ± 0.11	**0.79 ± 0.07**	0.53 ± 0.17
		(0.44 to 0.67)	(0.68 to 0.85)	
Myelin Feature	-	-	0.67	-
			(CI: 0.66 to 0.69)	

**Note**: Bold highlights the best results. Segmentation Regression+: segmentation regression plus lesion masks; Markov- GAM: GAM based on T2 signal intensity alone; Markov+ GAM: Markov GAM plus lesion masks.

#### 3.2.2 Quantitative results

The correlation between the GMRF myelin feature and histology myelin was 0.67 (top quartile: 0.78). It was 0.70 (top quartile: 0.78) for segmentation regression and 0.70 (top quartile: 0.78) for the Markov GAM (Table 2; Fig 6). The AUC analysis showed that the GMRF myelin feature had a stronger diagnostic ability than any of the regression models (p<10^−15^; Table 3). In addition, segmentation regression was more diagnostic than the GAMs in general (p = 10^−2^), where the Markov GAM was more diagnostic than Markov- GAM based on T2 intensity alone (p<10^−11^). With inclusion of manual lesion masks into the regression models (S2 Table), the GMRF myelin feature was still more diagnostic than the best segmentation regression+ (p<0.0010), but not significantly greater than the Markov GAM+. Further, according to the cross-validated R2 values (MAE models), the Markov GAM was significantly better than the Markov- GAM (p = 0.0031) and the linear GAM (p = 0.0040). With inclusion of lesion masks, the Markov+ GAM also tended to be better than without (p = 0.13; S5 Fig).

**Table 3:** AUC analysis for WM detection using the myelin predictions.

Model	Loss	Lesion Mask	Cohort(s) used	AUC	CI Low	CI High
Linear GAM	RMSE	n	all	0.832	0.822	0.832
	MAE	n	all	0.833	0.823	0.834
Segmentation Regression	RMSE	n	all	0.854	0.844	0.863
	MAE	n	all	0.856	0.846	0.866
Markov- GAM	RMSE	n	all	0.826	0.815	0.826
	MAE	n	all	0.827	0.816	0.827
Markov GAM	RMSE	n	all	0.844	0.835	0.855
	MAE	n	all	0.843	0.833	0.853
Myelin Feature	-	n	all	**0.883**	**0.874**	**0.891**
Segmentation Regression+	MAE	y	day 7	0.904	0.888	0.918
Markov+ GAM	MAE	y	day 7	0.910	0.895	0.924
Myelin Featuree	-	y	day 7	**0.919**	**0.905**	**0.932**

Note: Bold highlights the best results. CI: confidence interval, 95%. Segmentation Regression+: segmentation regression plus lesion masks; Markov- GAM: GAM based on T2 signal intensity alone; Markov+ GAM: Markov GAM plus lesion masks.

### 3.3 Estimation outcomes for the cellularity feature

**Fig 7:**
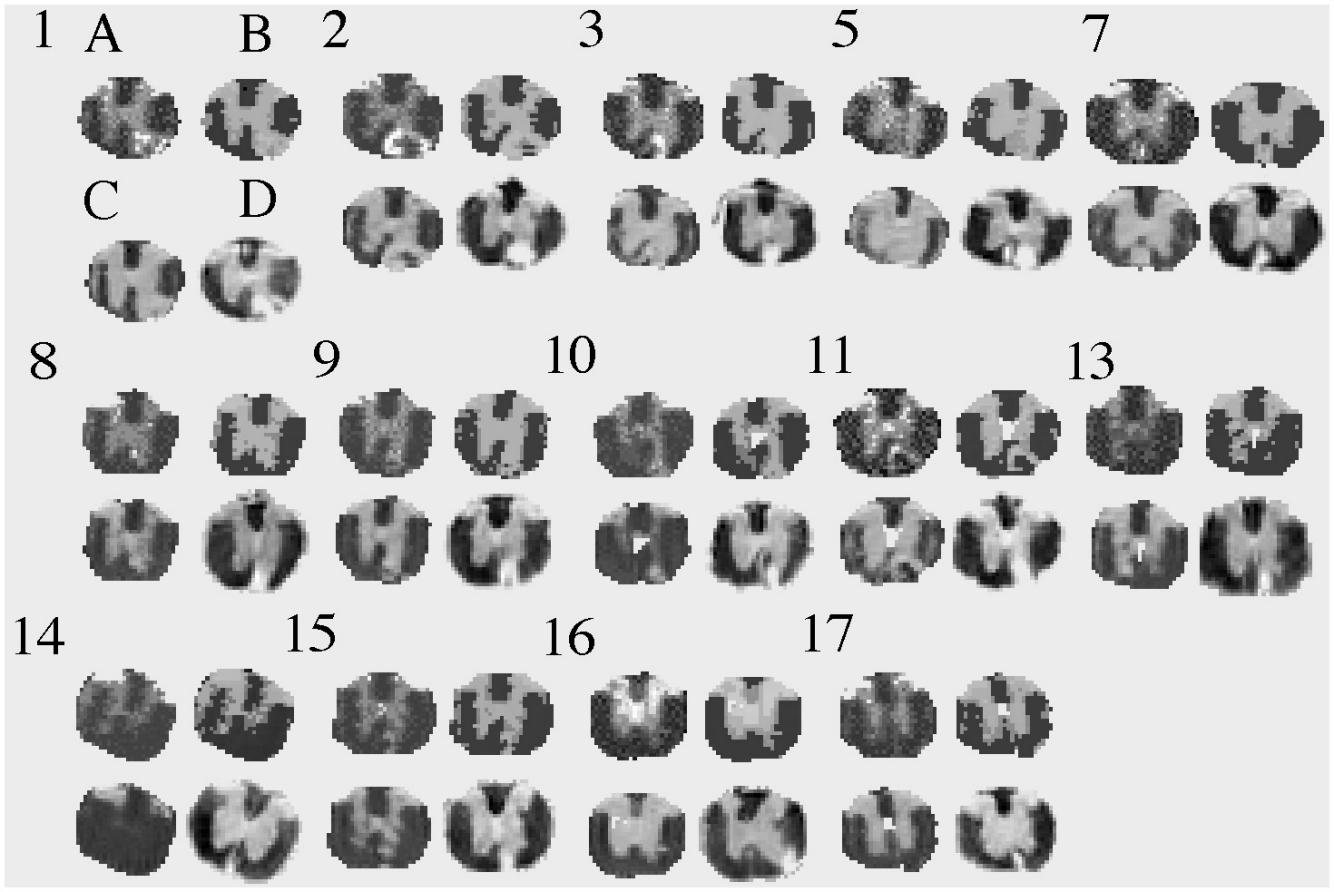
Cellularity predictions. Shown are the cellularity feature images learned from the MRI of the 14 mice that have histological correspondence, using our GMRF (A), segmentation regression associated with our GMRF (B), Markov GAM using a freely available software (C), and the histological standard (D). Note: The brighter the area, the greater the cellularity, except for the large lesion cores in mice 2 and 11, which show ‘dark signal’ paradoxically with the MRI methods.

The patterns and trends in the estimation of tissue cellularity were similar to those of myelin quantitatively. Overall, the correlation was 0.60 (top quartile: 0.70) between the predicted feature and histology cellularity using our GMRF, 0.69 (top quartile: 0.77) using segmentation regression, and 0.69 (top quartile: 0.77) using Markov GAM. Based on the R2 values (MAE models), the supervised models performed better with inclusion of the texture parameters than without (p = 0.0023 for Markov GAM). The Markov GAM was also better than the linear GAM (p = 0.0067), and tended to be better with inclusion of lesion masks than without (p = 0.25). Visually, the regression methods showed an under estimation in all lesions, and Markov GAM appeared to be the least accurate in mice 10 and 14 predictions (Fig 7 and Table 4).

**Table 4:** Mean ± standard deviation (min to max) cross-validated error and 95% confidence interval (min to max, last row) of cellularity model accuracies.

Model	Loss	R2	Pearson Correlation	Residual Correlation
Linear GAM	RMSE	0.31 ± 0.23	0.66 ± 0.11	0.84 ± 0.08
		(−0.36 to 0.51)	(0.48 to 0.84)	
	MAE	0.29 ± 0.30	0.66 ± 0.11	0.77 ± 0.09
		(−0.58 to 0.56)	(0.48 to 0.84)	
Segmentation Regression	RMSE	0.44 ± 0.17	0.69 ± 0.12	0.73 ± 0.08
		(0.08 to 0.66)	(0.44 to 0.85)	
	MAE	0.39 ± 0.24	0.69 ± 0.12	0.57 ± 0.09
		(−0.12 to 0.70)	(0.43 to 0.84)	
Segmentation Regression+	RMSE	**0.55 ± 0.05**	0.79 ± 0.03	0.58 ± 0.09
		(0.50 to 0.62)	(0.75 to 0.83)	
	MAE	0.52 ± 0.07	0.79 ± 0.03	**0.46 ± 0.13**
		(0.43 to 0.61)	(0.75 to 0.82)	
Markov-GAM	RMSE	0.35 ± 0.22	0.68 ± 0.12	0.82 ± 0.08
		(−0.26 to 0.59)	(0.45 to 0.86)	
	MAE	0.31 ± 0.29	0.67 ± 0.11	0.70 ± 0.10
		(−0.48 to 0.61)	(0.45 to 0.84)	
Markov GAM	RMSE	0.36 ± 0.26	0.68 ± 0.12	0.76 ± 0.08
		(−0.41 to 0.61)	(0.38 to 0.84)	
	MAE	0.36 ± 0.29	0.69 ± 0.12	0.70 ± 0.10
		(−0.47 to 0.66)	(0.42 to 0.85)	
Markov GAM+	RMSE	0.54 ± 0.07	0.79 ± 0.06	0.57 ± 0.14
		(0.49 to 0.64)	(0.72 to 0.86)	
	MAE	0.54 ± 0.07	**0.80 ± 0.06**	0.50 ± 0.18
		(0.49 to 0.66)	(0.73 to 0.87)	
Cellularity Feature	-	-	0.60	-
			(CI: 0.59 to 0.62)	

**Note**: Bold highlights the best results. Segmentation Regression+: segmentation regression plus lesion masks; Markov- GAM: GAM based on T2 signal intensity alone; Markov+ GAM: Markov GAM plus lesion masks.

### 3.4 Myelin and cellularity outcomes by time cohort

**Fig 8:**
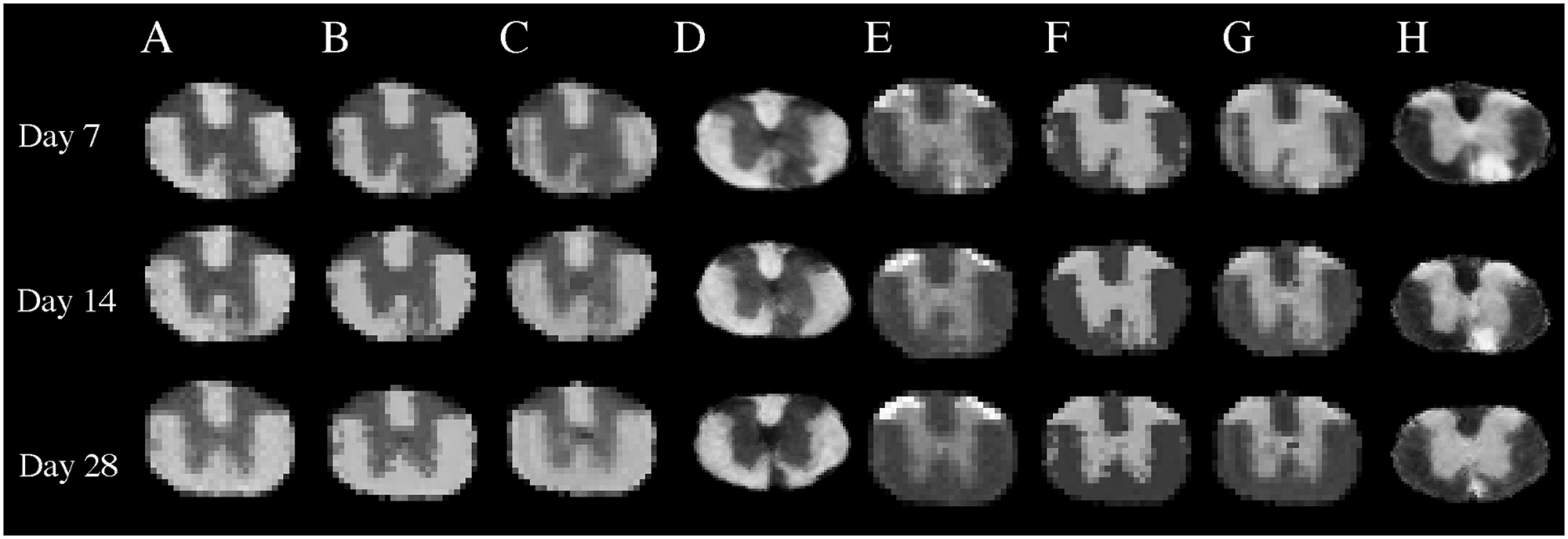
Myelin and cellularity predictions by cohort and time-point. Shown are the median outcomes for myelin obtained using our GMRF (A), segmentation regression associated with our GMRF (B), Markov GAM using freely available software (C), and the median histological myelin map (D), at days 7, 14, and 28. Similarly, images E-H demonstrate the median cellularity outcomes from GMRF, segmentation regression, Markov GAM, and histology, respectively, at the corresponding time-points. Note that the MRI predicted outcome in the lesion areas at day 28 are close to normal, while the histology myelin and cellularity in the corresponding sites remain abnormal.

Preliminary group-level analyses between time-points from all associated animals showed that there was a continual recovery of myelin estimates, and loss of cellularity estimates over time, consistent with the evolving pattern in histology. The MRI outcomes appeared to recover faster than histology outcomes in both myelin and cellularity, particularly in lesion cores, and this observation seemed to be present in all three of the MRI-based learning methods (Fig 8).

These observations were consistent with the quantitative outcomes as seen in the affected ventral white matter. The median myelin feature increased by 31 ± 12% and 78 ± 14% at days 14 and 28 from day 7, and cellularity decreased by 28 ± 5% and 46 ± 3%. The degree of changes in segmentation regression and Markov GAM were similar over time for both the tissue types. On the other hand, myelin content increased by 18 ± 8% and 47 ± 11%, and cellularity decreased by 15 ± 4% and 43 ± 3% in histology at days 14 and 28 from day 7, less than MRI changes. Correlation analyses showed a similar pattern, where the correlation coefficient between MRI and histology was much higher at days 7 and 14 than at day 28, but all values remained >0.8 (Tables 5 and 6).

**Table 5:** Cohort-level Pearson correlation (95% confidence interval) between myelin models and histology standards over time.

Model	Loss	Day 7 Cor	Day 14 Cor	Day 28 Cor	All Days Cor
Myelin Feature	-	**0.93** (0.91 to 0.94)	**0.93** (0.91 to 0.94)	0.84 (0.81 to 0.87)	**0.89** (0.88 to 0.90)
Segmentation Regression	RMSE	0.93 (0.91 to 0.94)	0.92 (0.91 to 0.94)	0.83 (0.80 to 0.86)	0.88 (0.87 to 0.89)
	MAE	0.93 (0.91 to 0.94)	0.92 (0.91 to 0.94)	0.84 (0.81 to 0.87)	0.88 (0.87 to 0.89)
Markov GAM	RMSE	0.91 (0.90 to 0.93)	0.92 (0.90 to 0.93)	0.84 (0.81 to 0.86)	0.87 (0.85 to 0.88)
	MAE	0.91 (0.90 to 0.93)	0.93 (0.92 to 0.94)	**0.85** (0.82 to 0.88)	0.87 (0.86 to 0.88)

**Table 6:** Cohort-level Pearson correlation (95% confidence interval) between cellularity models and histology standards over time.

Model	Loss	Day 7 Cor	Day 14 Cor	Day 28 Cor	All Days Cor
Cellularity Feature	-	0.89 (0.87 to 0.91)	0.82 (0.78 to 0.84)	0.70 (0.65 to 0.75)	0.81 (0.79 to 0.82)
Segmentation Regression	RMSE	0.91 (0.90 to 0.93)	0.89 (0.86 to 0.91)	0.82 (0.78 to 0.85)	0.87 (0.86 to 0.89)
	MAE	**0.92** (0.90 to 0.93)	**0.90** (0.88 to 0.92)	0.82 (0.79 to 0.85)	**0.88** (0.87 to 0.89)
Markov GAM	RMSE	0.88 (0.86 to 0.90)	0.87 (0.85 to 0.89)	0.82 (0.79 to 0.85)	0.85 (0.83 to 0.86)
	MAE	0.89 (0.87 to 0.91)	0.89 (0.87 to 0.91)	**0.84** (0.81 to 0.86)	0.86 (0.84 to 0.87)

## 4 Discussion

In this study, we developed a new unsupervised metric learning method for assessing myelin content using T2-weighted MRI and compared the outcomes against histology and 2 supervised approaches. In general, T2-weighted MRI is pathologically non-specific in MS [42]. However, based on a well-established de- and re-myelination model of MS, we discovered that Markov texture in T2 MRI was highly associated with the myelin integrity in mouse spinal cord. Further, integrating Markov texture into either unsupervised or supervised machine learning models helped extract valuable myelin content information from standard MRI.

Our prediction models were anatomically robust for most tissue groups segmented except for a few lesion cores. In particular, while lacking smoothness, our unsupervised GMRF features correlated strongly with histology, similar to the supervised models. In addition, the GMRF features were less susceptible to inter-subject variability than the GAM-based models, and more robust in identifying myelin rich structures than all supervised models as shown by the AUC. The two regression-based supervised learning approaches performed similarly, and both appeared to be better with use of: Markov texture, the MAE loss function, and lesion masks, than without. The non-significant difference between results with or without inclusion of lesion masks in the regression models may be due to the small number of animals (only 4), and the relatively small number of lesion pixels, deserving further verification. Notably, our GMRF model contributed to both unsupervised and supervised learning in the present study, where it performed metric learning directly in the former, and provided robust results enabling segmentation regression in the latter. Therefore, the GMRF can be a useful technique for both.

Compared to previously reported myelin predicting metrics using advanced MRI, our results were equally accurate. Prior R2 values were 0.49–0.61 (quantitative T1), 0.32–0.59 (quantitative T2), 0.67 (MWF), and 0.2–0.71 (MTR) in MS brain, and 0.56 (MWF) in rat sciatic nerve [43]. Our R2 values were similar to those with T1 and T2, and likely better with inclusion of manual lesion masks in supervised learning. In fact, our best fit (mouse 5, MAE segmentation regression) achieved a much higher R2 than all of the above reports, even without the use of lesion masks. In addition, based on the same experiment, a prior study has investigated the utility of high angular resolution diffusion imaging (HARDI), which is hypothesized to be more specific to myelin and axonal changes than the traditional diffusion tensor imaging metrics (e.g. radial and axial diffusivity). In that study, a HARDI measure termed orientation dispersion index showed a considerable increase with demyelination and recovery with remyelination in mouse spinal cord, with continuing increase of myelin content as reflected by the neurite density index [12]. Our cellularity predictions performed similarly to but somewhat worse than that of myelin in the present study, and appeared to be more specific when including lesion masks, as indicated by the high cellularity versus myelin contrast in lesions and the GM. It is worth noting that at least 5 of the 6 studies done previously used ROI-based analyses in histology-MRI correlation, which may have overestimated the R2 values relative to the pixel-wise approach done in our present study.

Tissue segmentation was a critical intermediate step in this study. Our GMRF segmentation was 15% more accurate than the initiating GMM, indicating the importance of texture information. In contrast, while also using a MRF, FAST segmentation performed 6% worse than the GMM. Technically, the FAST differs from our GMRF in three key respects: 1) FAST is optimized using iterative conditional modes, 2) it does not allow *a priori* tissue proportions, and 3) it uses a Markov hidden label field [14] whereas we used the Markov property directly on source MRI signal intensity. The increased performance of GMRF was likely due to our use of the optimizer, which applied increased iterations, and the temperature parameter that prevented convergence to a local minimum. The use of *a priori* tissue proportions might also help explain the difference, although our *ad hoc* tests suggested that the main contribution of including tissue proportions was preventing empty labels. Notably, the FAST showed a higher sensitivity to CB than the GMRF, although at the cost of a much greater false positive rate from GM to CB simultaneously. One potential explanation is that the two CB populations (SG and lesion pixels) were primarily similar in staining identity rather than texture. The GMRF is much stronger texture-dependence, making it more sensitive to the differences between SG and lesions than the FAST, and hence a lower detectability to the CB as a group. Our overall outcomes also indicate that proper lesion detection in MRI is critical for improving model accuracy. While we applied manual lesion masks, there is extensive literature on the topic of lesion identification using unsupervised approaches [20], which however is out of the scope of the present study.

The main challenge of our GMRF model was in classifying lesions with a relatively large core that contained unusually dark signal intensity on T2 MRI. Most of these lesion pixels were classified as GM rather than CB, which likely also propagated errors to the estimation of GMRF features and segmentation regression. This was most obvious in a few day 7 lesions, which were mistakenly characterized as having increased myelin and decreased cellularity before mask use. The exact cause of low T2 signal intensity within the lesions remained unclear. As our lesions were induced by the injection of a chemical toxin, the decreased signal could be due to the following factors: mechanical damage, the impact of degraded myelin (debris), and an iron susceptibility effect associated with the myelin-forming cells and inflammatory cells [44]. Given the purpose of the current study, degraded myelin was not evaluated (eriochrome cyanine detects only healthy myelin). Previous work [45] reported the same problem when segmenting lesions using a similar model. Nonetheless, such intra-lesion signal loss on T2-weighted MRI is not common in the MRI of humans with MS, so the potential limitations related to the lesion core are not expected to have a major effect on future testing of our method in a clinical setting. Moreover, with application of lesion masks in supervised learning, our results demonstrate the feasibility to achieve reasonable estimates of myelin and cellularity features for these lesions.

Using co-registered longitudinal data, we also explored the changes in myelin and cellularity at a cohort level. By ‘averaging’ (median) outcomes across individuals per time-point, this analysis allowed to minimize random errors while preserving anatomy, including the lesions. We observed a significant increase in Pearson correlation between our models and histology in both myelin and cellularity. Over time, there was a reduction of cellularity in lesions, and a recovery of WM in lesion periphery (decreasing lesion size) in all model outcomes. Moreover, the MRI-based myelin and cellularity estimates seemed to recover faster than histology, as confirmed both visually and quantitatively: there was a near-complete resolution of lesions by day 28 in MRI measures in contrast to histology, and a sudden drop in Pearson correlation between them at day 28 versus days 7 and 14. These observations may suggest that the MRI features reflect more physical changes than lipid content (blue) and cell nuclei (red) as highlighted in histology [23]. T2-weighted MRI is sensitive primarily to water content, but that may also depend on the presence of macromolecules such as lipids, and proton-proton interactions [46]. We suspect that due to the recovery differences between MRI and histology metrics, the MRI models could no longer distinguish tiny lesion areas from the WM when the amount of macromolecules dropped below a certain threshold such as that occurred at day 28.

We note a few limitations in this study. First, the number of animals per time-point was small, limiting broad conclusions. Second, the inherent challenges in histological analysis may have affected the results. The presence of tears and bubbles in stained images is not uncommon, requiring pre-processing to optimize segmentation accuracy. Moreover, it is difficult to wash the blue staining out of nuclei without also washing the stain off of myelin [23], causing the nuclei to be stained with both red and blue (purple). While almost unavoidable in this type of studies, it may have created ambiguity in the ground truth. Variations in staining density is another common issue, for which we performed image normalization to compensate. Third, accurate MRI and histology correspondence has been a long-term challenge, potentially adding uncertainties to the analyses. One issue comes from the resolution differences between the modalities. In this study, we applied several image preparation steps to improve alignment, including 2 iterations of image co-registration. Another issue relates to the *ex vivo* nature of histology versus *in vivo* MRI. However, immediately before MRI, we applied high-dose anesthesia to keep the animals still, making optimal acquisition of images possible. In addition, all animals had their tissue samples prepared immediately after imaging for histology, which allowed best preservation of tissue integrity. Further, histological validation of MRI measures has been one of the standard approaches in both animal and human studies, and our current observations are highly consistent with results from prior studies using the same model [3, 12]. Fourth, our image contrast in MRI is relatively modest. Our in-house experiments show that images with better quality would result in greater accuracy in both segmentation and feature estimation with the GMRF, consistent with the rule-of-thumb stating that good contrast facilitates good segmentation [14]. Therefore, our current results seemed to set a low threshold for the requirement of the quality of MRI scans, and our findings are rather conservative. Finally, we did not have a true longitudinal cohort, limiting systemic time series analyses. However, cohort-level analysis following strict longitudinal co-registration served as an alternative approach, particularly for this established animal model, which is highly predictable in both disease course and lesion location [21, 47]. In the future, we seek to extend our GMRF model to a full Gaussian random field or beyond [36, 48], replace it with a multi-scale approach [49], or modify it with outlier detection [20, 50] to improve performance. Notably, our approach was purposefully designed as stepwise and modular, to enable further validation and extension. In addition, we plan to test alternative metrics, such as an exact geodesic distance [51] to compare with the Mahalanobis distance, and test additional imaging sequences [45] and anatomical regions (e.g. the brain) to further validate our method.

## 5 Conclusions

Using standard T2-weighted MRI and a robust model for de- and re-myelination, we implemented a novel GMRF algorithm for both unsupervised and supervised learning of myelin and cellularity. Our MRI myelin feature performed similarly well to the literature that used advanced MRI, and to supervised learning approaches. Likewise, our GMRF segmentation outperformed a common FSL FAST model by >20% in accuracy. Collective results suggest that image analysis using adequate mathematical models has the ability to extract valuable myelin information from standard MRI. With further confirmation, the GMRF method can be similarly applied to interrogate myelin characteristics concealed in clinical MRI of humans with MS or similar diseases. This will be achievable as long as there is a dedicated tissue group that has high cellularity in the images, such as focal lesions, which in fact are the hallmark pathology of many demyelinating disorders. Integrating such novel approaches in clinical practice can advance the use of standard MRI, provide new information about myelin injury and repair, and add new quantitative measures in disease diagnosis and management.

## Supporting information

S1 Fig GMRF MRI segmentation simulations. Shown are both the original T2 MRI (A) and the simulated images using the GMRF parameters (B) estimated for each animal (1–18). Row indicates time cohort: day 7 (row 1), day 14 (row 2) or day 28 (row 3). The simulated image for mouse 14 appears to be an outlier.
(TIF)

S2 Fig Segmentation regression versus GAM residuals. Shown are the residual images from segmentation regression using the MAE as a loss function following myelin (A) and cellularity (C) predictions, in comparison with the corresponding residual images from the Markov GAM on myelin (B) and cellularity (D). Higher visibility of the anatomical structures (e.g. the ‘H’ shaped GM region) indicates poorer fitting of the models.
(TIF)

S3 Fig Mean (RMSE) versus median (MAE) myelin regression. Shown are segmentation regression using the RMSE (A) and MAE (B), and Markov GAM regression using RMSE (C) and MAE (D). In both cases, using MAE showed better contrast between tissue types than using RMSE.
(TIF)

S4 Fig Mean (RMSE) versus median (MAE) cellularity regression. Shown are segmentation regression using the RMSE (A) and MAE (B), and Markov GAM regression using RMSE (C) and MAE (D). In both cases, using MAE showed better contrast between tissue types than using RMSE.
(TIF)

S5 Fig Regression results with inclusion of lesion masks. Shown are the myelin (a, top) and cellularity (b, bottom) predictions for the four day 7 mice (numbers) that contain the largest lesions. The demonstrations include the original T2 MRI overlaid with manual lesion masks (A, red outline), prediction results from the supervised, cross-validated models using segmentation regression (B) and Markov GAM (C), and histological standards (D).
(TIF)

S1 Table Noteworthy T2 MRI segmentation parameters (mean ± sd). (DOCX)

S2 Table Mean Absolute Error (MAE) segmentation regression estimates following cross-validation along with use of lesion masks (mean ± sd). (DOCX)

S1 File Hidden label field estimation. (DOCX)

S2 File Gibbs sampling simulation procedure. (DOCX)

S1 Data (CSV)

S2 Data (CSV)

S3 Data (CSV)
