# Supplementary figures and images for: A metric learning method for estimating myelin content based on T2-weighted MRI from a de- and re-myelination model of multiple sclerosis
Source: PLoS One. 2021 Apr 5;16(4):e0249460. doi: 10.1371/journal.pone.0249460 (PMC8021181; doi:10.1371/journal.pone.0249460)

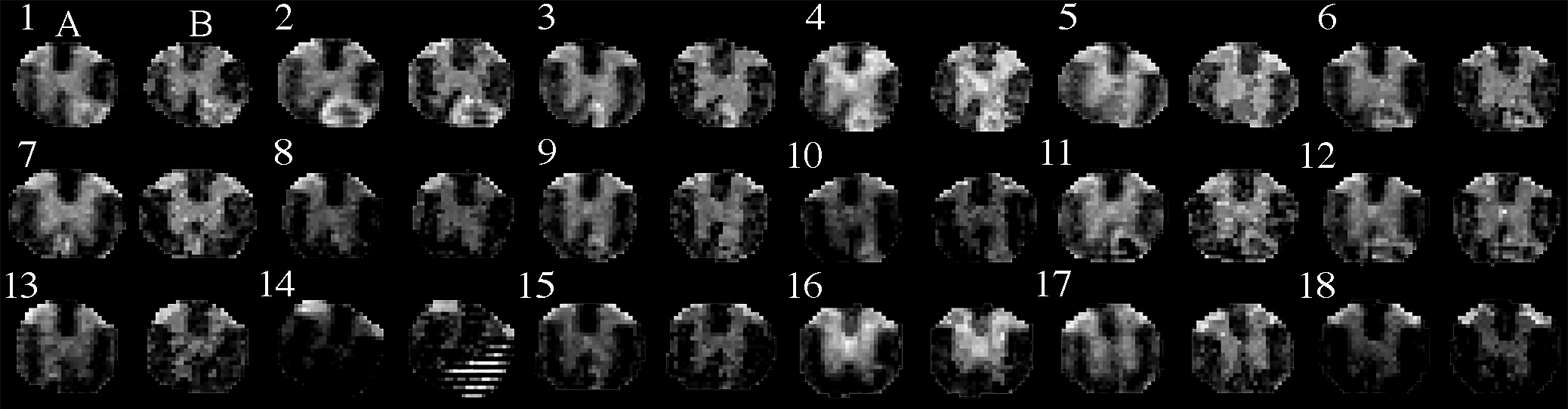

Supplement: S1 Fig — Shown are both the original T2 MRI (A) and the simulated images using the GMRF parameters (B) estimated for each animal (1–18). Row indicates time cohort: day 7 (row 1), day 14 (row 2) or day 28 (row 3). The simulated image for mouse 14 appears to be an outlier. (TIF) [file pone.0249460.s001.tif]

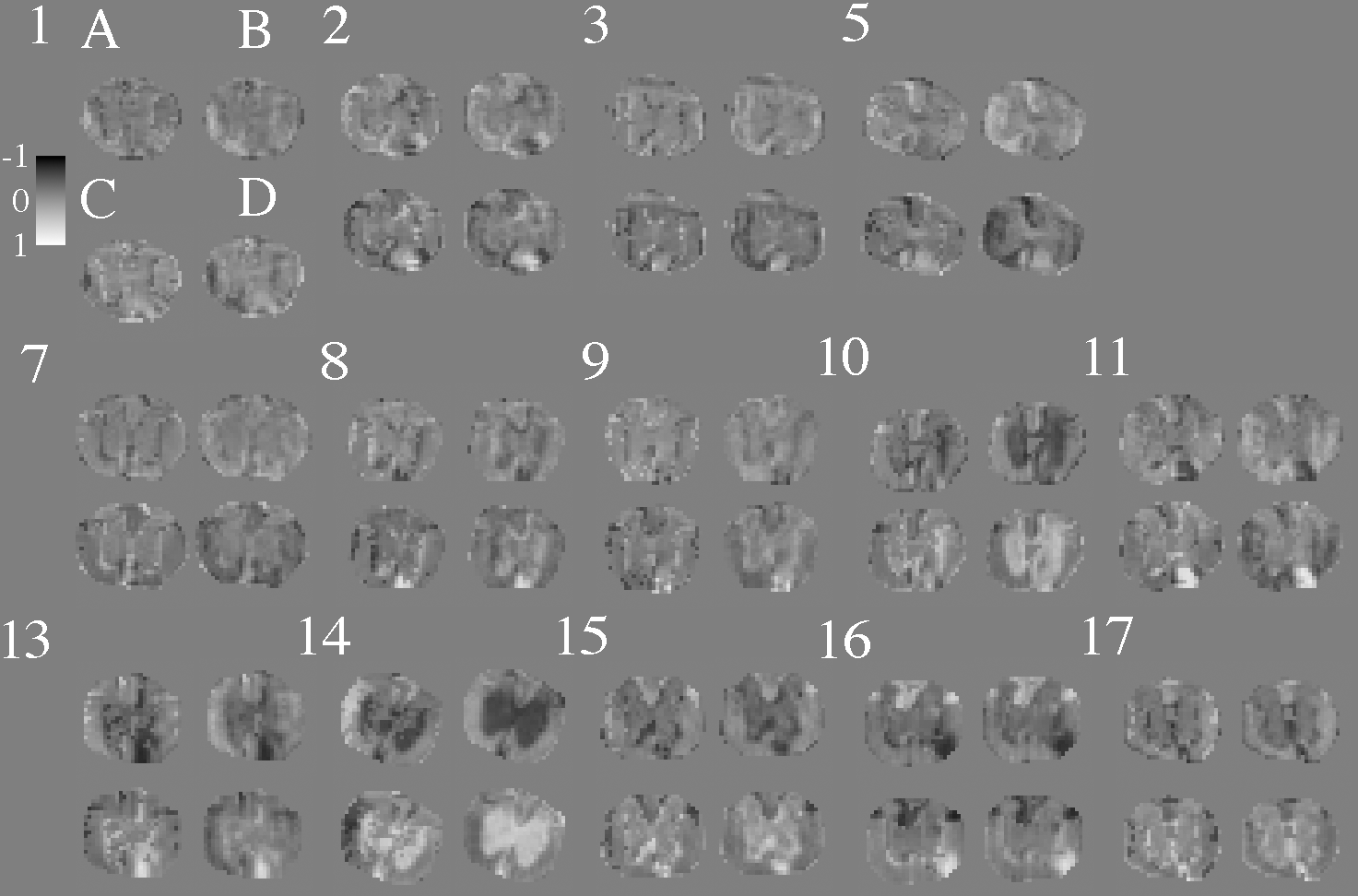

Supplement: S2 Fig — Shown are the residual images from segmentation regression using the MAE as a loss function following myelin (A) and cellularity (C) predictions, in comparison with the corresponding residual images from the Markov GAM on myelin (B) and cellularity (D). Higher visibility of the anatomical structures (e.g. the ‘H’ shaped GM region) indicates poorer fitting of the models. (TIF) [file pone.0249460.s002.tif]

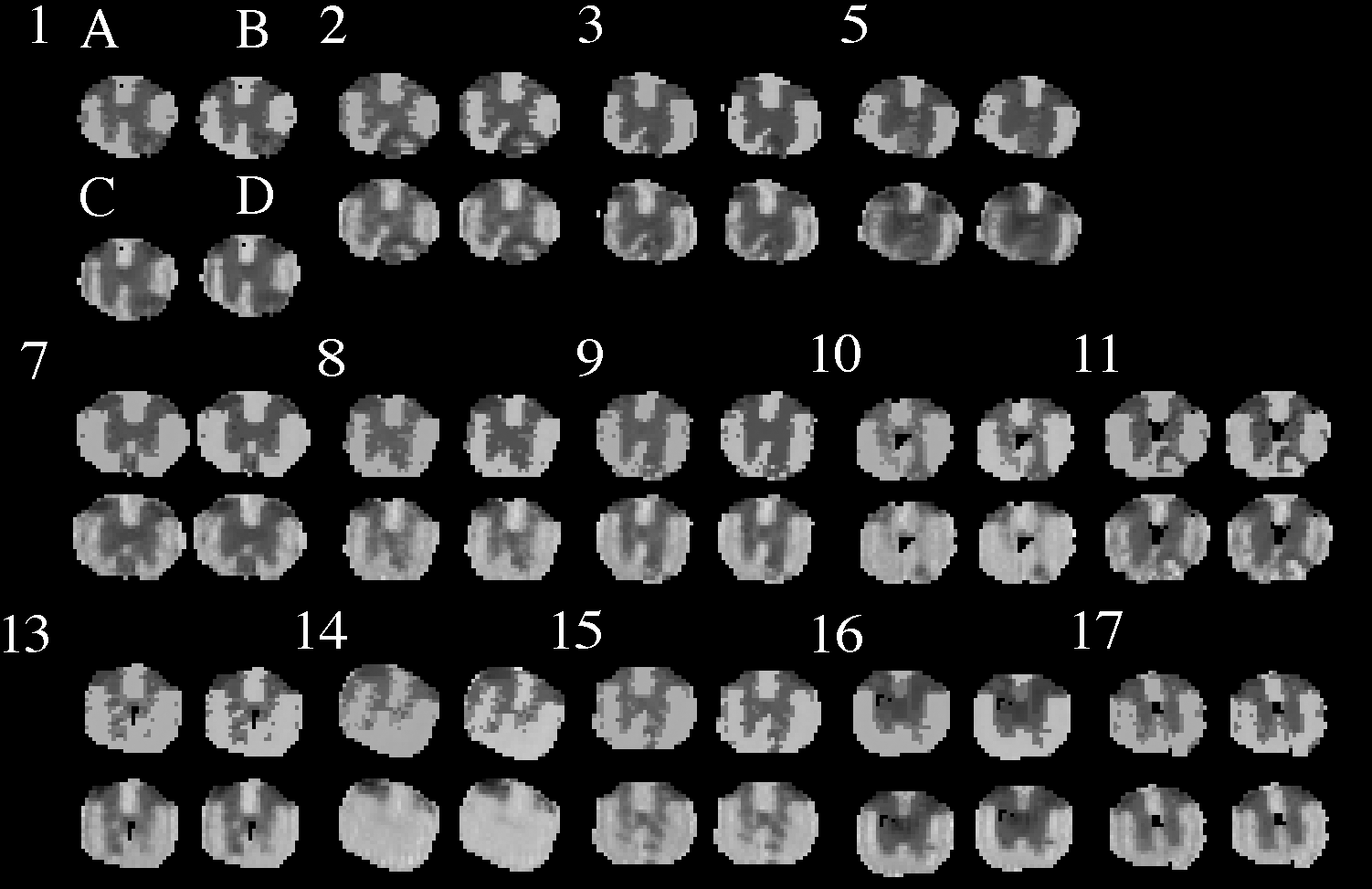

Supplement: S3 Fig — Shown are segmentation regression using the RMSE (A) and MAE (B), and Markov GAM regression using RMSE (C) and MAE (D). In both cases, using MAE showed better contrast between tissue types than using RMSE. (TIF) [file pone.0249460.s003.tif]

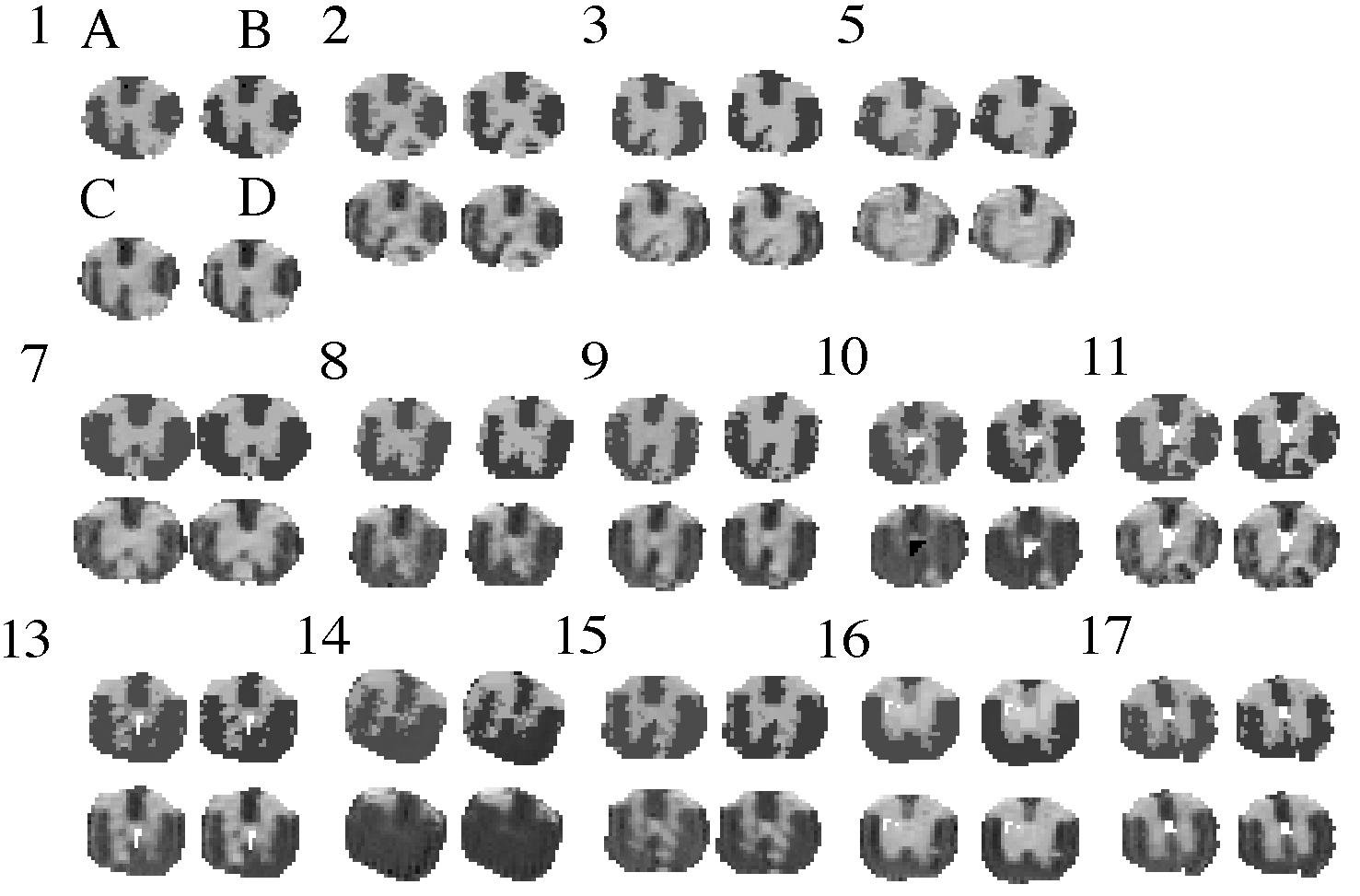

Supplement: S4 Fig — Shown are segmentation regression using the RMSE (A) and MAE (B), and Markov GAM regression using RMSE (C) and MAE (D). In both cases, using MAE showed better contrast between tissue types than using RMSE. (TIF) [file pone.0249460.s004.tif]

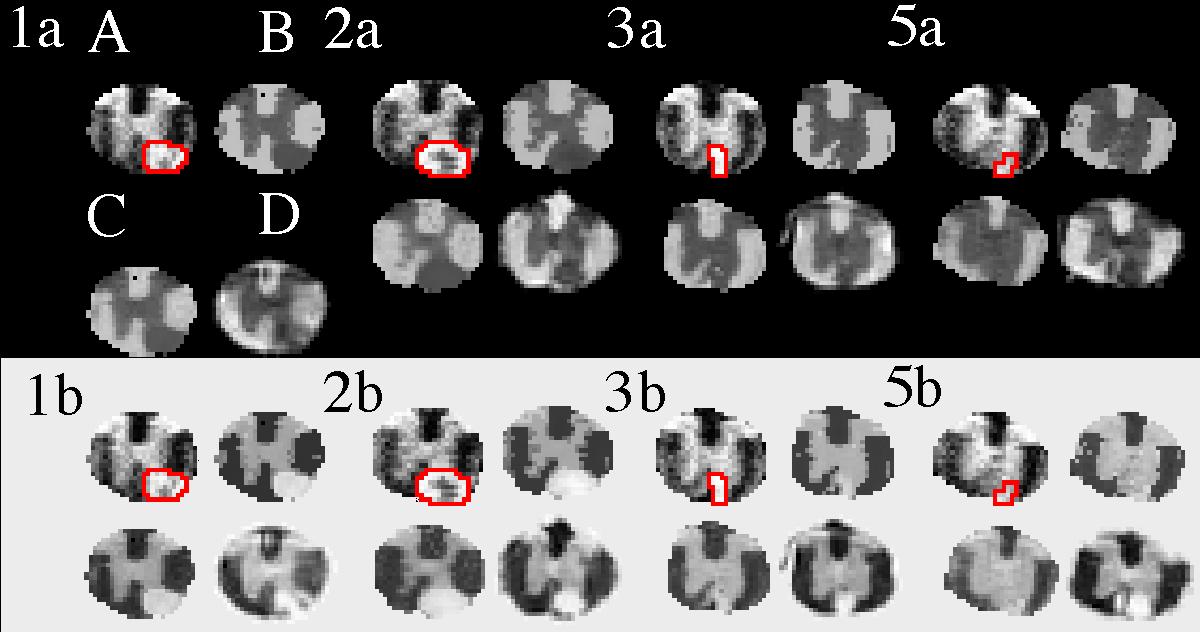

Supplement: S5 Fig — Shown are the myelin (a, top) and cellularity (b, bottom) predictions for the four day 7 mice (numbers) that contain the largest lesions. The demonstrations include the original T2 MRI overlaid with manual lesion masks (A, red outline), prediction results from the supervised, cross-validated models using segmentation regression (B) and Markov GAM (C), and histological standards (D). (TIF) [file pone.0249460.s005.tif]
