# Supplementary material for: A metric learning method for estimating myelin content based on T2-weighted MRI from a de- and re-myelination model of multiple sclerosis
Source: PLoS One. 2021 Apr 5;16(4):e0249460. doi: 10.1371/journal.pone.0249460 (PMC8021181; doi:10.1371/journal.pone.0249460)
Supplement: S1 Table — (DOCX) [file pone.0249460.s006.docx]

**S1 Table: Noteworthy T2 MRI segmentation parameters (mean ± sd).**

| Label | $\boldsymbol{\mu}$ | $\boldsymbol{\beta}_{\boldsymbol{00}}$ | $\boldsymbol{\beta}_{\boldsymbol{10}}$ | $\boldsymbol{\beta}_{\boldsymbol{01}}$ |
| --- | --- | --- | --- | --- |
| WM | $0.574\pm0.042$ | $9.88\pm2.56\cdot{10}^{-4}$ | $0.299\pm0.071$ | $0.251\pm0.090$ |
| PV | $0.677\pm0.053$ | $2.65\pm1.02\cdot{10}^{-4}$ | $0.103\pm0.043$ | $0.082\pm0.042$ |
| GM | $0.757\pm0.056$ | $5.02\pm1.35\cdot{10}^{-4}$ | $0.227\pm0.087$ | $0.100\pm0.077$ |
| CB | $0.889\pm0.027$ | $8.67\pm4.38\cdot{10}^{-4}$ | $0.323\pm0.146$ | $0.027\pm0.196$ |
| BG | $0\pm0$ | $1\pm0\cdot{10}^{-30}$ | $0\pm0$ | $0\pm0$ |

Note: Reported here are the first four parameters from the Gaussian Markov random field for each of the five segmentation classes.
