# Supplementary material for: A metric learning method for estimating myelin content based on T2-weighted MRI from a de- and re-myelination model of multiple sclerosis
Source: PLoS One. 2021 Apr 5;16(4):e0249460. doi: 10.1371/journal.pone.0249460 (PMC8021181; doi:10.1371/journal.pone.0249460)
Supplement: S2 Table — (DOCX) [file pone.0249460.s007.docx]

**S2 Table: Mean absolute error (MAE) segmentation regression estimates following cross-validation along with use of lesion masks (mean** $\boldsymbol{\pm}$ **sd).**

| **Tissue** | **Blue (Myelin)** | | **Red (Cellularity)** | |
| --- | --- | --- | --- | --- |
|  | **Intercept** | **Slope** | **Intercept** | **Slope** |
| SG | $0.22\pm0.22$ | $0.04\pm0.25$ | $0.75\pm0.13$ | $0.03\pm0.14$ |
| GM | $0.782\pm0.037$ | $-0.566\pm0.051$ | $0.32\pm0.15$ | $0.46\pm0.20$ |
| PV | $2.05\pm0.19$ | $-2.28\pm0.28$ | $-1.15\pm0.43$ | $2.43\pm0.64$ |
| WM | $0.854\pm0.087$ | $-0.20\pm0.16$ | $0.13\pm0.11$ | $0.16\pm0.2$0 |
| Lesion | $0.183\pm0.053$ | $0.158\pm0.058$ | $1.210\pm0.040$ | $-0.422\pm0.055$ |
