# Supplementary material for: A metric learning method for estimating myelin content based on T2-weighted MRI from a de- and re-myelination model of multiple sclerosis
Source: PLoS One. 2021 Apr 5;16(4):e0249460. doi: 10.1371/journal.pone.0249460 (PMC8021181; doi:10.1371/journal.pone.0249460)
Supplement: S1 File — (DOCX) [file pone.0249460.s008.docx]

**Hidden Label Field Estimation**

We derived the closed expression for the proposal distribution:

| $p\left( Z_{il}=1\vert Z_{-il},x,Q,\mu\right)$ | (A.1) |
| --- | --- |

We factored the probabilities using Bayes’ theorem and the Markov property (Rue and Held, 2005) to:

| $p\left( Z_{il}=1 \vert Z_{-il},x,Q,\mu\right)=\frac{1}{\Pi}p\left( x_{i} \vert\mathcal{N}_{i},Q,\mu,Z_{il}=1,Z_{-il} \right)p\left( \mathcal{N}_{i} \vert Q,\mu,Z_{-il} \right)$  $\cdot p\left( Q,\mu\vert Z_{il}=1,Z_{-il} \right)p\left( Z_{-il} \vert Z_{il}=1 \right)p\left( Z_{il}=1 \right)$ | (A.2) |
| --- | --- |

where $\Pi$ is the normalization constant, and $\mathcal{N}_{i}$ is the neighbourhood of $x_{i}$. We assumed that the hidden label field was independent:

| ${p(Z\_}_{il}\left\vert Z_{il} \right)={p(Z\_}_{il})$ | (A.3) |
| --- | --- |

We also used the *ad hoc* approximation:

| $p\left( Q,\mu\vert Z_{il}=1,Z_{-il} \right)\approx p\left( Q,\mu\vert Z_{-il} \right)$ | (A.4) |
| --- | --- |

This allowed us to simplify the expression considerably because any term that does not explicitly depend on the current label membership $Z_{il}$ will factor out of the normalization sum and cancel with the numerator. The final estimator was:

| $p\left( Z_{il}=1 \vert Z_{-il},x,Q,\mu\right)\approx\frac{1}{\Pi}p\left( x_{i} \vert\mathcal{N}_{i},Q,\mu,Z_{il}=1,Z_{-il} \right)p\left( Z_{il}=1 \right)$ | (A.5) |
| --- | --- |

which required knowledge of only the two probability densities in the product on the right-hand side. The final result was exponentiated to the inverse temperature to facilitate simulated annealing.
