# Supplementary material for: A metric learning method for estimating myelin content based on T2-weighted MRI from a de- and re-myelination model of multiple sclerosis
Source: PLoS One. 2021 Apr 5;16(4):e0249460. doi: 10.1371/journal.pone.0249460 (PMC8021181; doi:10.1371/journal.pone.0249460)
Supplement: S2 File — (DOCX) [file pone.0249460.s009.docx]

**Gibbs Sampling Simulation Procedure**

1. Generate white noise with mean $\mu$ and variance $\mathrm{diag}\left( Q \right)^{-\frac{1}{2}}$.
2. Perform Gibbs sampling using Eq. (5) via image coding.
3. After a burn-in period of 3 iterations, estimate the parameters of the simulated image using least-squares at each iteration.
4. Stop when the least-squares parameters converge below a pre-set tolerance on the standard deviation between steps (5%) or when the maximum number of iterations is reached (5).
